# Supplementary material for: Machine learning-guided deconvolution of plasma protein levels
Source: Mol Syst Biol. 2025 Oct 9;21(12):1822–44. doi: 10.1038/s44320-025-00158-6 (PMC12672695; doi:10.1038/s44320-025-00158-6)
Supplement: Supplementary file 23 — Expanded View Figures [file 44320_2025_158_MOESM23_ESM.pdf]

Expanded View Figures

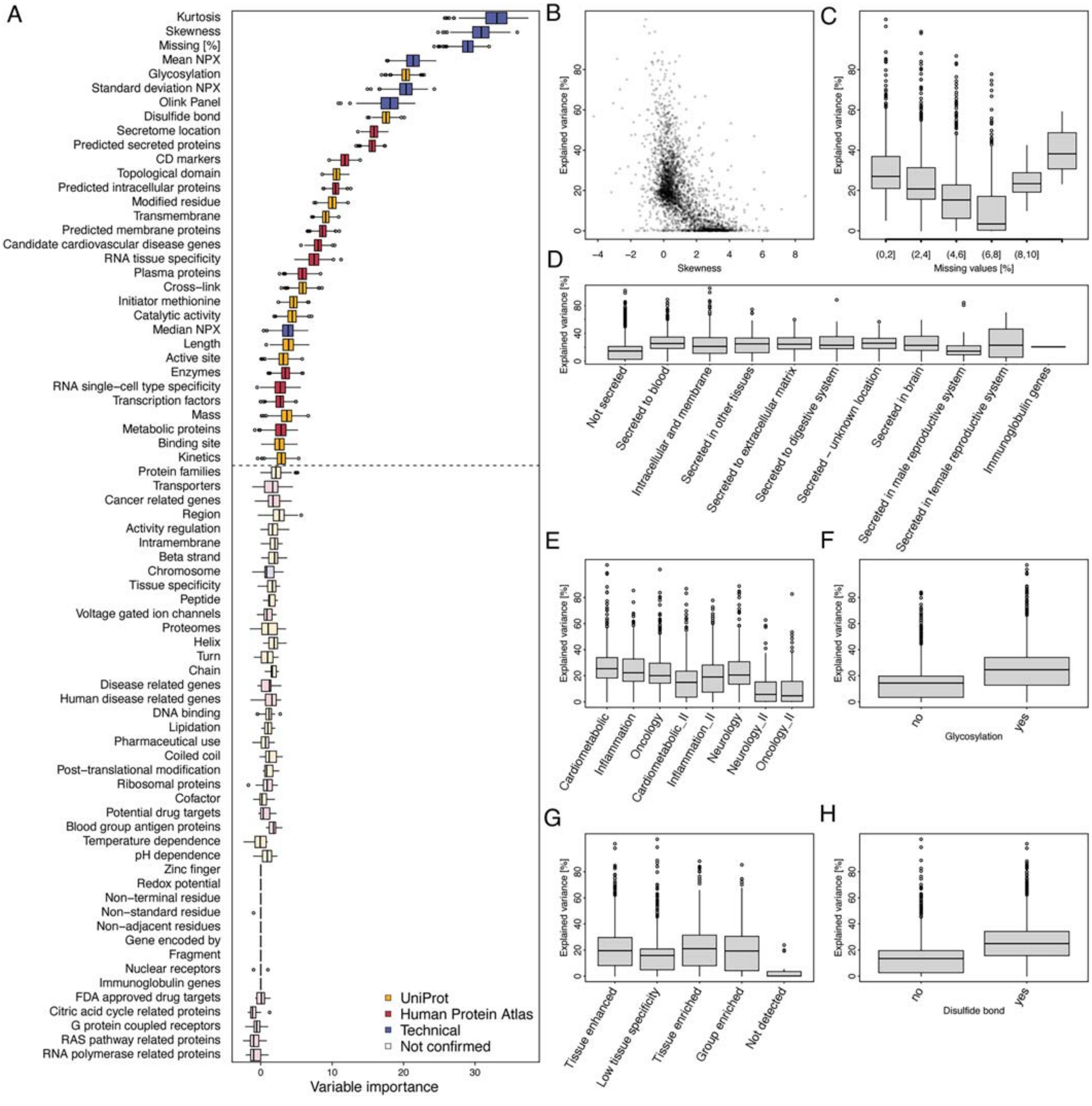

Figure EV1. Summary of protein and assay characteristics associated with the variance explained achieved in plasma protein levels.

(**A**) Variable importance of protein and assay characteristics based on Boruta feature selection, predicting the variance explained achieved for each protein target. Boxplots indicate the distribution of the variable importance across 500 iterations. Darker colours indicate features passing corrected statistical significance ($p < 0.01$). Analysis included a total of 2853 protein targets for which at least one feature that explained variation in plasma levels could be identified. Boxplots were drawn using default options: lower whiskers = 25th percentile − 1.5 x interquartile range; upper whiskers = 75th percentile + 1.5 x interquartile range; centre = 50th percentile (median); lower box bound = 25th percentile; upper box bound = 75th percentile; minima and maxima represent the most extreme values and are plotted as outliers if exceeding whiskers. (**B**) Scatterplot opposing the skewness of individual plasma protein distributions with the variance explained. (**C–H**) Variance explained according to different criteria deemed important by the Boruta feature selection. Displayed and explained variance values for a total of 2853 protein targets for which at least one feature that explained variation in plasma levels could be identified. Boxplots were drawn using default options: lower whiskers = 25th percentile – 1.5 x interquartile range; upper whiskers = 75th percentile + 1.5 x interquartile range; centre = 50th percentile (median); lower box bound = 25th percentile; upper box bound = 75th percentile; minima and maxima represent the most extreme values and are plotted as outliers if exceeding whiskers.

© The Author(s)

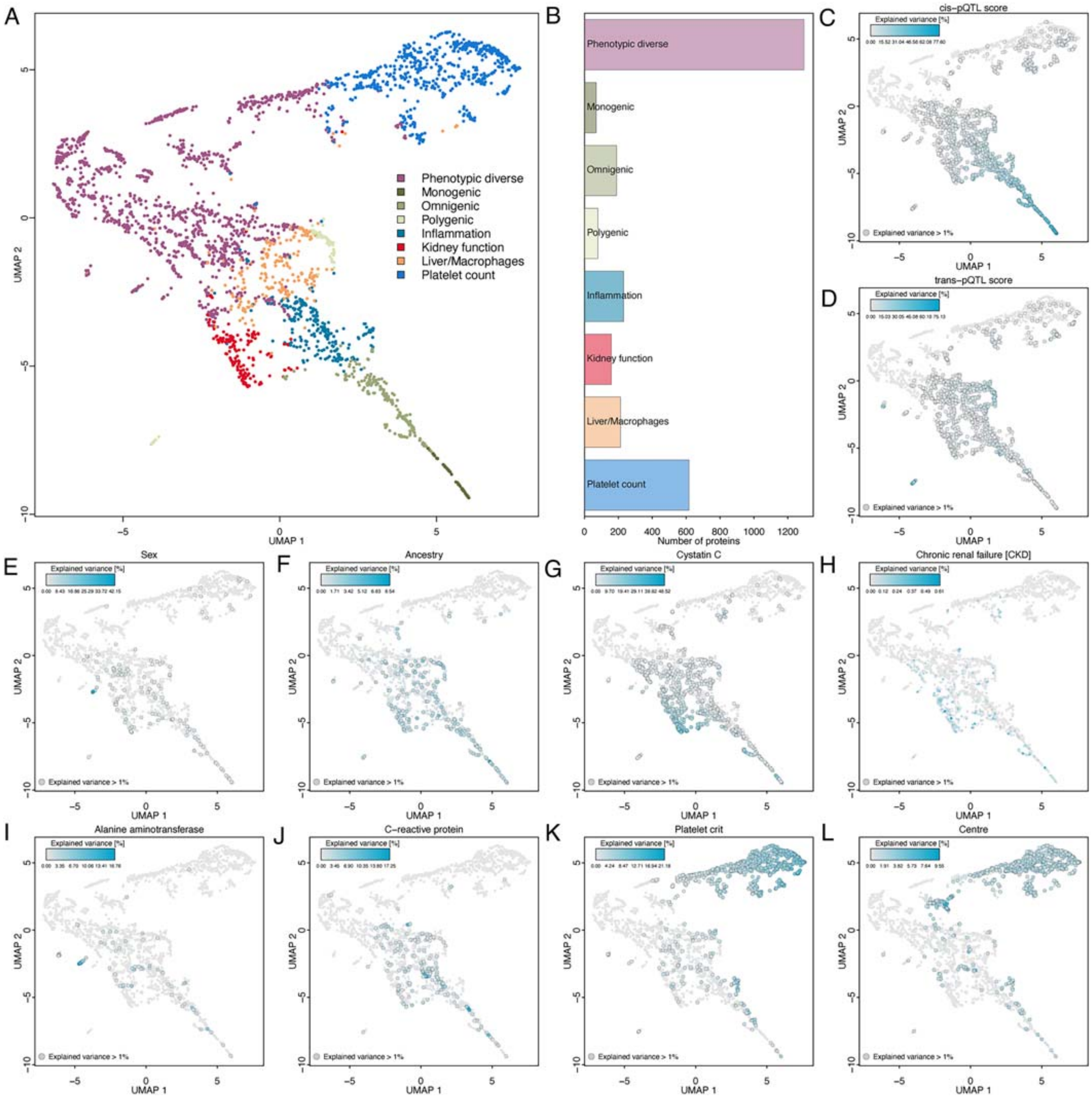

Figure EV2. Major foundations of plasma protein variation.

(**A**) Uniform manifold approximation and projection (UMAP) mapping of the variance explained matrix across 2853 protein targets for which we identified at least one feature explaining the variance in plasma levels. Each protein has been assigned a cluster based on k-means clustering and is coloured accordingly. (**B**) Number of protein targets included in each cluster. (**C–L**) Same UMAP plot but coloured according to the variance explained by the factor given on top of each plot. Proteins with strong contributions (>1%) are highlighted. pQTL protein quantitative trait loci.

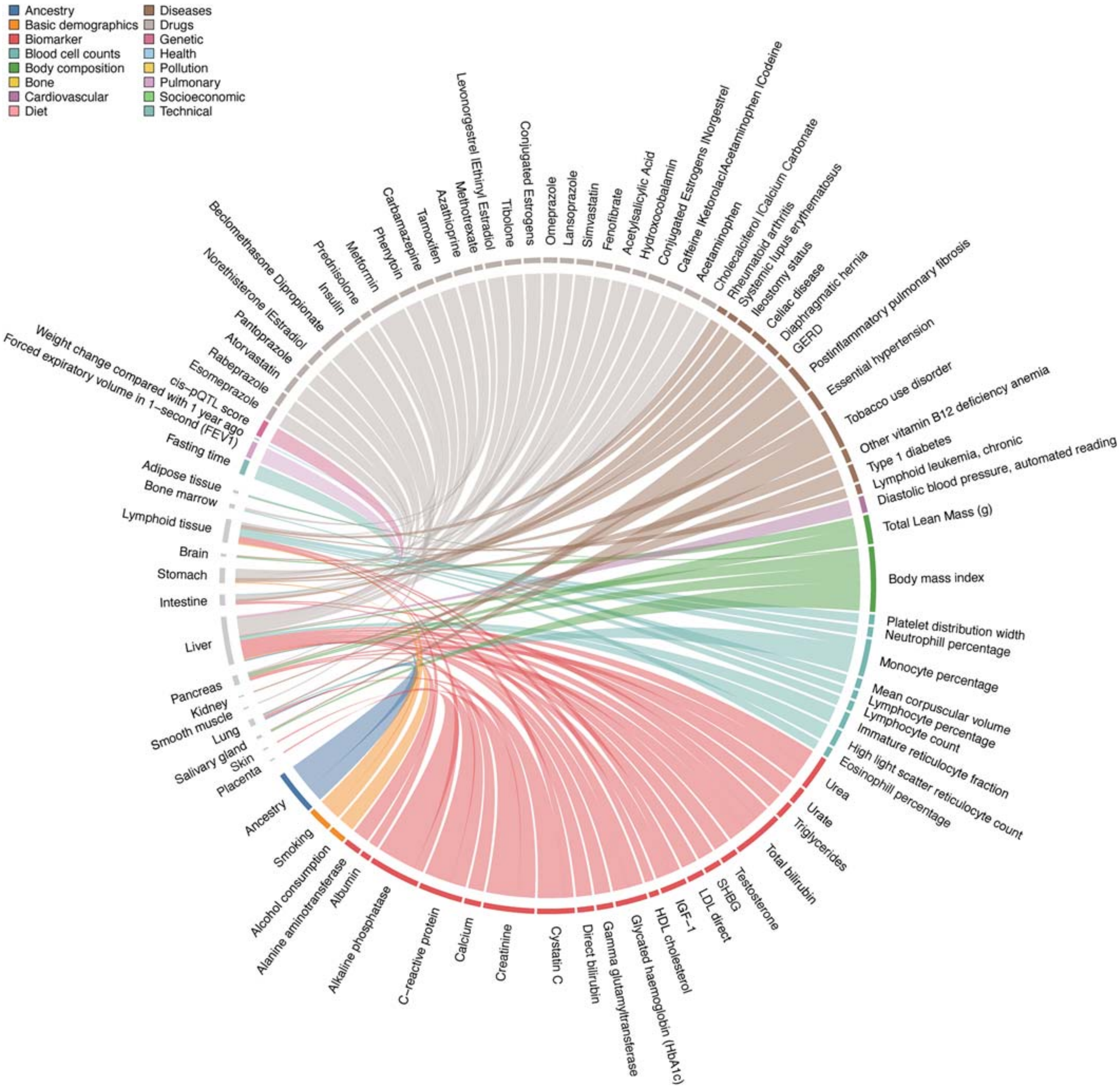

Figure EV3. Chord diagram of phenotype-associated protein signature enrichment among protein-coding genes with enhanced tissue expression.

Each line represents a significant enrichment ($p < 6.9 \times 10^{-6}$) of proteins associated with a given participant characteristic among protein-coding genes with enhanced expression in a given tissue. Enhanced expression estimates were derived from the Human Protein Atlas. Corresponding statistics can be found in Dataset EV7. Colouring was done according to phenotype categories as introduced in Fig. 1 in the main text.

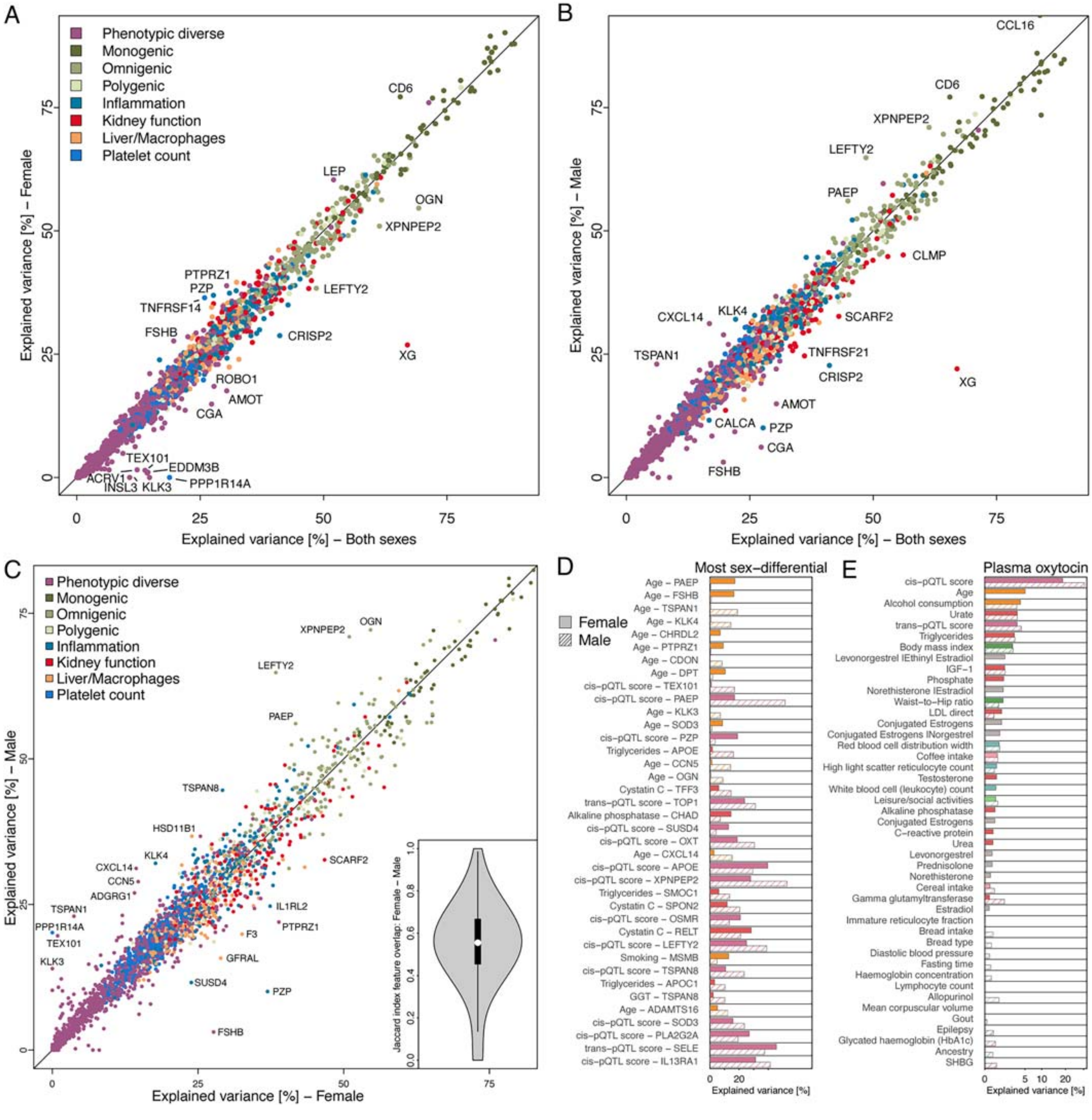

Figure EV4. Summary of sex-differential feature selection and contribution.

(**A**, **B**) Scatterplots comparing the variance explained achieved for plasma protein levels among the entire UK Biobank population (x-axis) compared to what was achieved in females (left) and males (right) alone. Proteins that deviated the most (>4 s.d.) were annotated. (**C**) Comparison of cumulative variance explained when stratifying the UK Biobank population by sex ($n = 23,601$ females, $n = 20,055$ males). Proteins are coloured by cluster assignments as introduced in Fig. 1. Proteins with strong differences are annotated with gene names. The inlet depicts the distribution of the Jaccard index of overlapping features for the same protein across the sexes. Boxplot was drawn using default options: lower whiskers = 25th percentile − 1.5 x interquartile range; upper whiskers = 75th percentile + 1.5 x interquartile range; centre = 50th percentile (median); lower box bound = 25th percentile; upper box bound = 75th percentile; minima and maxima represent the most extreme values and are plotted as outliers if exceeding whiskers. (**D**) Protein—Feature combinations with significant evidence for sex-differential effects ($p < 9.0 \times 10^{-7}$). Only combinations with a difference of more than 10% are shown. Sex-differential effects were assessed using an interaction term in a linear regression model. (**E**) Individual variance explained estimates for plasma oxytocin levels by sex, ordered by the variance explained among females. Proteins in b and c are coloured according to feature categories as introduced in Fig. 1. Variance explained was derived from a multivariable linear regression model as the partial R^2.

 © The Author(s)

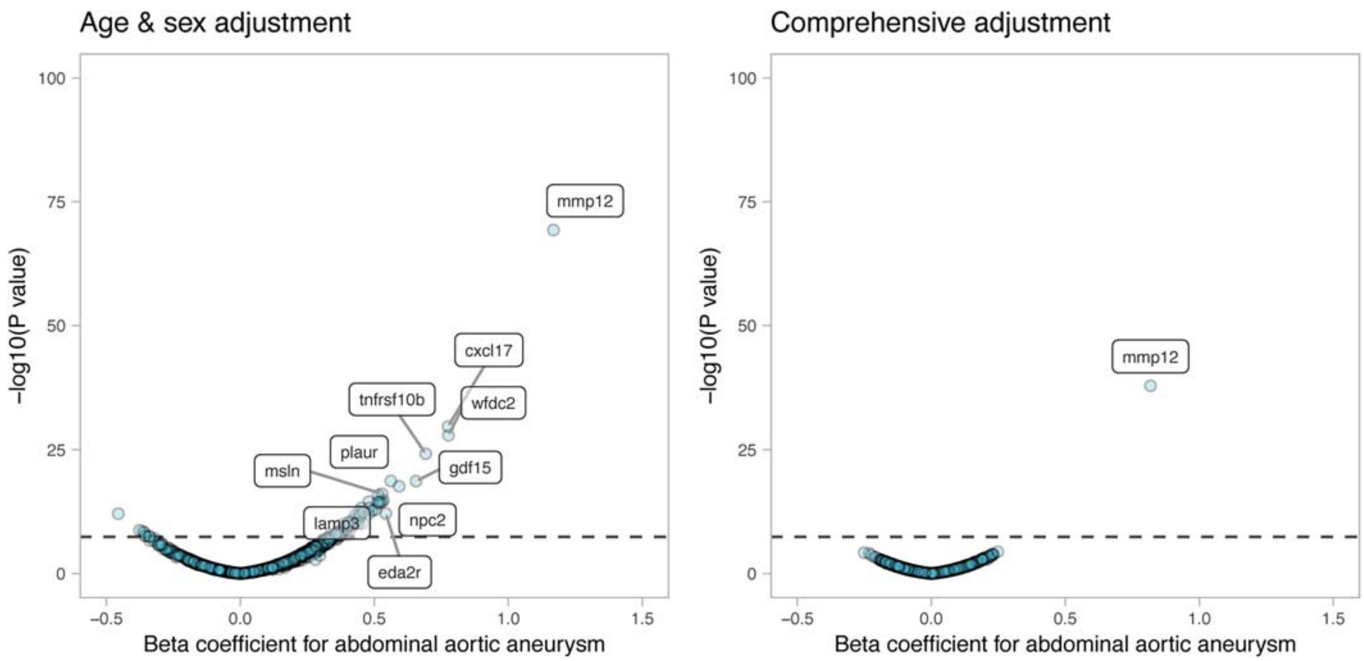

Figure EV5. Plasma protein biomarkers associated with the onset of abdominal aortic aneurysm.

Volcano plots for protein–abdominal aortic aneurysm associations, with adjustment for age and sex (left panel), or comprehensive adjustment based on results from the feature selection algorithm for each protein target (right panel). Association statistics were derived from Cox-proportional hazard models.

© The Author(s)

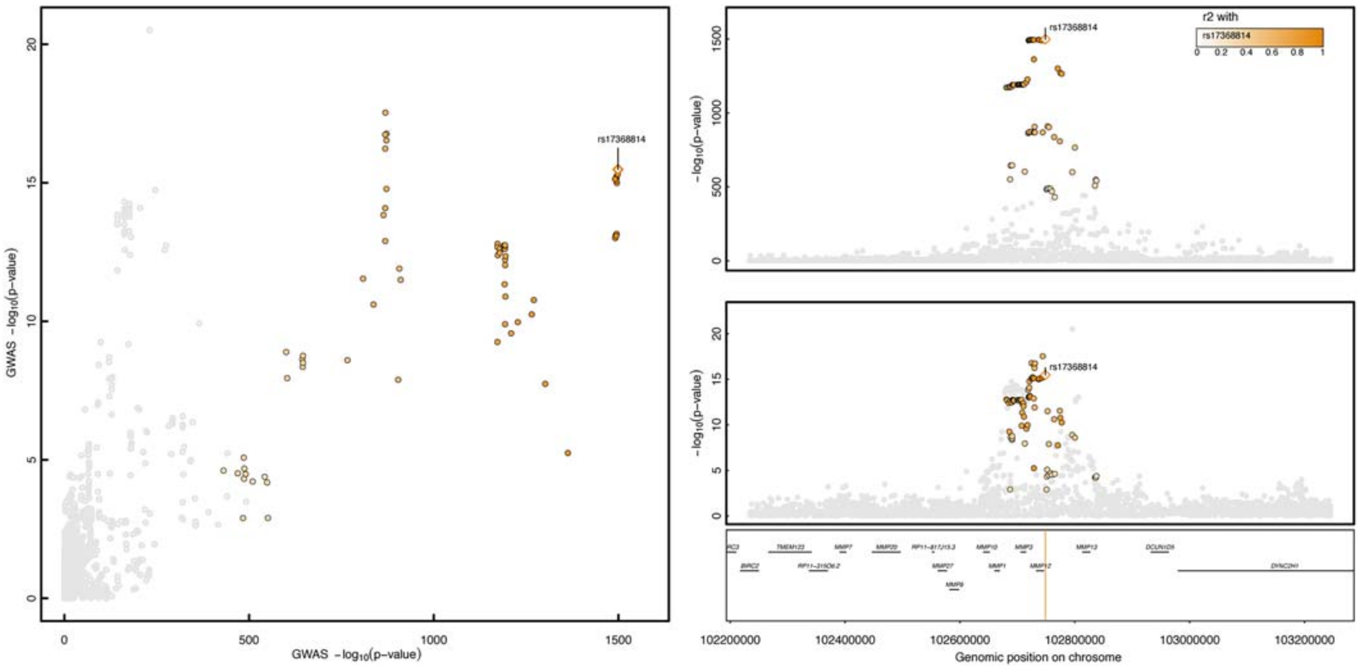

Figure EV6. Genetic variation at the *MMP12* locus is associated with the onset of abdominal aortic aneurysm.

Regional association plot of the *MMP12* locus for MMP12 protein plasma levels (top) and abdominal aortic aneurysm (AAA, bottom). One single-nucleotide polymorphism (rs17368814) has been prioritised as a shared genetic signal. Summary statistics from logistic regression models for AAA are publicly available from the AAAgen consortium and summary statistics from linear regression models for MMP12 plasma levels are based on UK Biobank. Colouring indicates linkage disequilibrium to the lead variant of the same colour code. Probability for a shared genetic signal (PP-H_4) is given as $PP_{rs17368814} = 97.5\%$.

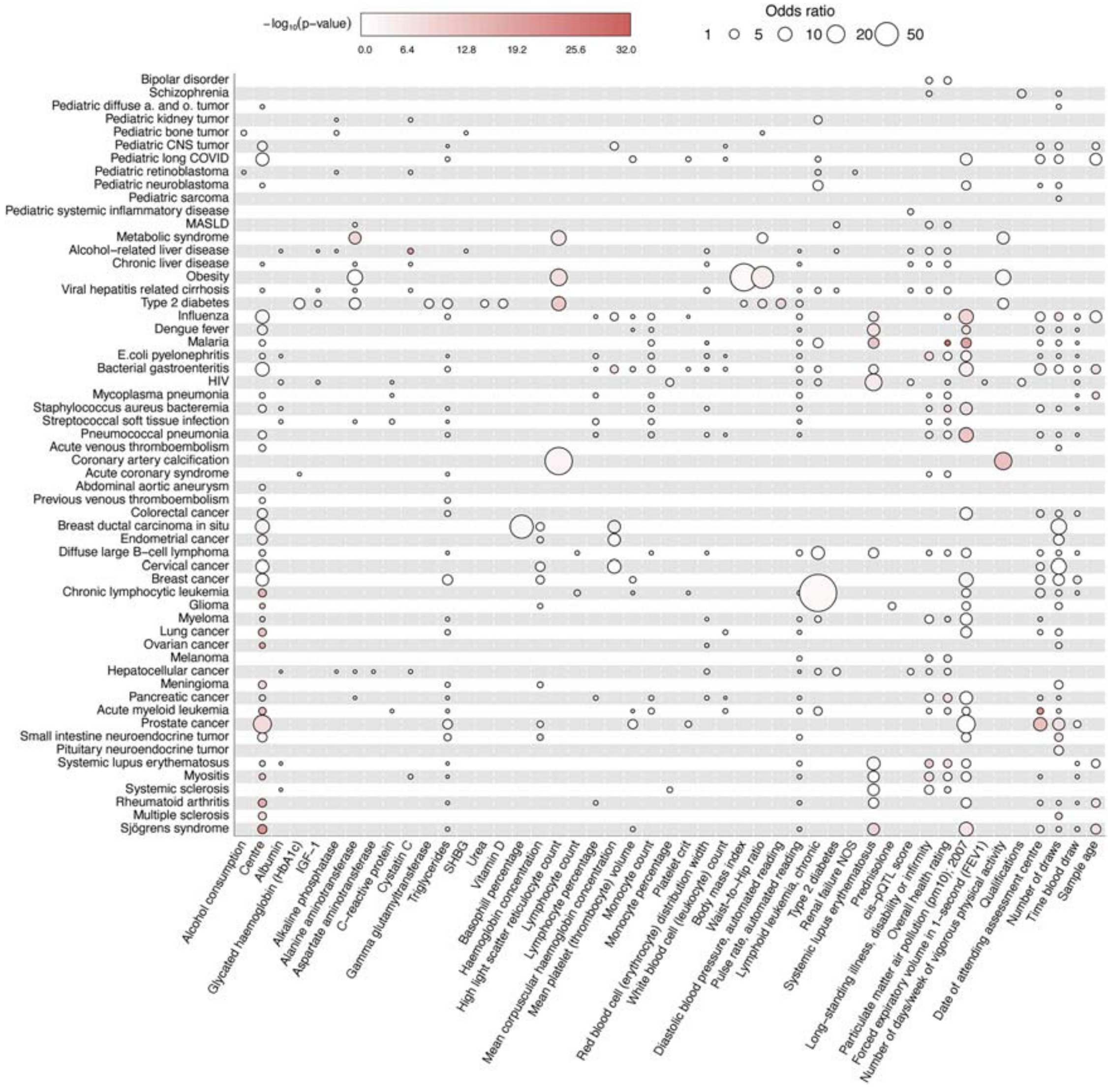

Figure EV7. Phenotype enrichment among differentially expressed plasma proteins between a random set of controls and different patient groups from the blood protein atlas section of the Human Protein Atlas.

Each row refers to a protein signature significantly differential (corrected *p* value <0.05 and |log-fold change| >0.5) in the plasma of diseased patients. Each column refers to phenotype-associated protein signature from the protein atlas that was significantly ($p < 4.7 \times 10^{-6}$) enriched among the respective disease–protein signatures. Significant findings are highlighted by dots, with colour representing transformed *p* values and size reflecting odds ratios. Enrichment was done using Fisher's exact test.
